# Complete Genome Sequences of Four Different *Bordetella* sp. Isolates Causing Human Respiratory Infections

**DOI:** 10.1128/genomeA.01080-16

**Published:** 2016-10-06

**Authors:** Michael R. Weigand, Yanhui Peng, Vladimir Loparev, Dhwani Batra, Katherine E. Bowden, Pamela K. Cassiday, Jamie K. Davis, Taccara Johnson, Phalasy Juieng, Christine E. Miner, Lori Rowe, Mili Sheth, M. Lucia Tondella, Margaret M. Williams

**Affiliations:** Centers for Disease Control and Prevention, Atlanta, Georgia, USA

## Abstract

Species of the genus *Bordetella* associate with various animal hosts, frequently causing respiratory disease. *Bordetella pertussis* is the primary agent of whooping cough and other *Bordetella* species can cause similar cough illness. Here, we report four complete genome sequences from isolates of different *Bordetella* species recovered from human respiratory infections.

## GENOME ANNOUNCEMENT

*Bordetella* species are associated with a variety of hosts, where they are often the etiologic agents of disease. Most notably, *Bordetella pertussis* causes whooping cough (pertussis) but related species *B. parapertussis* and *B. holmesii* can cause similar pertussis-like illness in humans (1–5). *B. bronchiseptica* and *B. holmesii* have also been reported to cause infection at multiple human body sites in addition to the respiratory tract (6–10). Through Enhanced Pertussis Surveillance, the Centers for Disease Control and Prevention occasionally receives isolates of *Bordetella* species other than *B. pertussis*. Here, we report four complete genome sequences from clinical isolates of *B. parapertussis* (H904), *B. bronchiseptica* (I328), *B. holmesii* (H903), and unclassified *Bordetella* sp. (H567) which were all recovered from patients with respiratory infection.

Whole-genome shotgun sequencing was performed using a combination of the PacBio RSII (Pacific Biosciences, Menlo Park, CA), Illumina HiSeq/MiSeq (Illumina, San Diego, CA), and Argus (OpGen, Gaithersburg, MA) platforms as described previously (11). Briefly, genomic DNA libraries were prepared for PacBio sequencing using the SMRTbell template prep kit 1.0 and Polymerase binding kit P4, while Illumina libraries were prepared using the NEB Ultra library prep kit (New England Biolabs, Ipswich, MA). *De novo* genome assembly of filtered reads was performed using the Hierarchical Genome Assembly Process (HGAP, v3, Pacific Biosciences) (12) with at least 140× coverage. The resulting consensus sequences were determined with Quiver (v1), manually checked for circularity, and then re-ordered to start at the coding region for glucose-inhibited cell division protein A (*gidA*), consistent with available genome sequences of *Bordetella* species. To ensure accuracy, assemblies were confirmed by comparison to BamHI and KpnI restriction digest optical maps using the Argus system (OpGen) with MapSolver (v.2.1.1, OpGen) and further “polished” by mapping Illumina HiSeq PE-100 or MiSeq PE-300 reads using CLC Genomics Workbench (v8.5, CLC bio, Boston, MA). Final assemblies were annotated using the NCBI automated Prokaryotic Genome Annotation Pipeline (PGAP).

Isolate and assembly characteristics are summarized in Table 1. As expected, the four genomes varied in size, G+C content, the number of predicted protein-coding genes, and structural arrangement. Using the genome sequence, isolate H567 could only be classified to the genus *Bordetella* and did not match any named species or group according to either 16S (13, 14) or *nrdA* (15) gene sequences. Phylogenetically, H567 appeared distant from the “classic” bordetellae and more closely related to proposed *Bordetella* species isolated from respiratory samples of cystic fibrosis patients (14) but distinct from the neighboring genus *Achromobacter*. Isolate H567 also behaved more similarly to species proposed by Vandamme et al. (14) and was oxidase positive, catalase positive, urease negative, nitrate negative, and motile, consistent with its phylogenetic placement.

**TABLE 1:** Characteristics of *Bordetella* sp. respiratory isolates and genome assemblies

Taxonomy	Isolate	Specimen type	U.S. state	Yr of isolation	Genome size (bp)	G+C (%)	CDSs^a^	Accession no.
*B. parapertussis*	H904	Unknown	NY	2012	4,773,708	68.1	4,161	CP016342
*B. bronchiseptica*	I328	Throat swab	MN	2012	5,077,489	68.4	4,630	CP016431
*B. holmesii*	H903	Unknown	NY	2012	3,694,378	62.3	3,378	CP016341
*Bordetella* sp.	H567	NP^b^ swab	CA	2010	5,532,470	66.1	4,630	CP012334

a CDSs, coding sequences.

b NP, nasopharyngeal.

Several *Bordetella* species have been recovered from human respiratory tract specimens, including putative novel species like isolate H567. The availability of complete genome sequences from species other than *B. pertussis* should aid research of how the broader *Bordetella* genus causes respiratory illness in humans.

### Accession number(s).

The complete genome sequences have been deposited at DDBJ/EMBL/GenBank under the accession numbers listed in Table 1. The versions described in this paper are the first versions.
